# Does Cardiopulmonary Bypass Affect Outcomes in Nephrectomy with Level III/IV Caval Thrombectomy for Renal Cell Carcinoma?

**DOI:** 10.3390/curroncol32120671

**Published:** 2025-11-29

**Authors:** John V. Dudinec, Alireza Ghoreifi, Justin Refugia, Sriram Deivasigamani, Michael Ivey, Alexandra E. Hunter, Farshad S. Moghaddam, Abigail R. Benkert, Joseph J. Fantony, Adam R. Williams, Ankeet Shah, Michael R. Abern

**Affiliations:** 1 Department of Urology, Duke University Medical Center, Durham, NC 27705, USA; john.dudinec@duke.edu (J.V.D.); justin.refugia@duke.edu (J.R.); michael.c.ivey@duke.edu (M.I.); alexandra.hunter@duke.edu (A.E.H.); joseph.fantony@duke.edu (J.J.F.); ankeet.shah@duke.edu (A.S.); 2 Department of Urology, University of Washington, Seatle, WA 98195, USA; fsheym@uw.edu; 3 Division of Thoracic and Cardiovascular Surgery, Department of Surgery, Duke University Medical Center, Durham, NC 27705, USA; abigail.benkert@duke.edu (A.R.B.); adam.r.williams@duke.edu (A.R.W.)

**Keywords:** inferior vena cava, renal cell carcinoma, thrombectomy, radical nephrectomy

## Abstract

Radical nephrectomy with inferior vena cava (IVC) thrombectomy is a technically complex procedure. Cardiopulmonary bypass (CPB) is frequently employed for managing high-level thrombi, yet its impact on surgical outcomes remains uncertain. This study evaluated the outcomes of radical nephrectomy with level III/IV thrombectomy with or without CPB. We retrospectively reviewed records of patients with renal cell carcinoma and level III/IV (Mayo classification) thrombi who underwent open radical nephrectomy and IVC thrombectomy at our center between January 2000 and December 2023. Perioperative and survival outcomes were compared between patients in the CPB and non-CPB groups. Multivariable regression identified clinical factors associated with all-grade complications and survival. Primary and secondary outcomes were 90-day complications and overall survival. Fifty-seven patients were included: 30 (53%) in the CPB group and 27 (47%) in the non-CPB group. Within 90 days, overall complication and mortality rates were 49% and 10.5%; no statistically significant differences were observed between groups. In multivariable models, CPB was not independently associated with 90-day complications (odds ratio [OR] 0.55, 95% CI 0.13–2.12, *p* = 0.4) or overall survival (hazard ratio [HR] 1.34, 95% CI 0.65–2.78, *p* = 0.41). In our cohort, we did not find CPB use to independently influence perioperative complications or survival outcomes in radical nephrectomy with level III/IV IVC thrombectomy.

## Simple Summary

Renal cell carcinoma with upper-level (level III/level IV) tumor thrombus of the inferior vena cava is rare, and surgical management remains a mainstay of treatment. Cardiopulmonary bypass (CPB) is often used in these patients for hemodynamic management; however, concerns remain regarding its associated risks. Previous work has questioned whether CPB increases complication rates or worsens long-term outcomes for patients undergoing nephrectomy with upper-level thrombectomy. In this single-center study, we found no difference in complication rates or overall survival between patients managed with and without CPB. Although comparative data on surgical techniques are lacking, our findings add to the growing evidence that CPB use is not independently associated with complications or survival in patients undergoing nephrectomy with level III/IV thrombectomy.

## 1. Introduction

Venous tumor invasion is present in 4–10% of renal cell carcinomas (RCC), with fewer than 1% of patients having tumor thrombus extension at or above the hepatic veins (level III/level IV tumor thrombus) [1]. The prognosis of RCC with tumor thrombus is grim, with a reported median survival of 5 months in an untreated population [2], though aggressive surgical management with complete tumor resection has been shown to provide long-term survival benefit and oncologic control in nonmetastatic patients [3]. Surgical management of these higher-level thrombi is technically complex and requires meticulous hemodynamic control. Cardiopulmonary bypass (CPB) is often employed in these cases, though data vary as to whether CPB has an impact on morbidity or mortality [4,5,6,7,8]. Various non-CPB techniques for management of higher-level thrombi [9,10,11] have been described, though comparative data among surgical techniques are lacking.

To address this question, we evaluated surgical and survival outcomes of patients undergoing open radical nephrectomy with IVC thrombectomy for RCC with level III/IV caval thrombus, comparing cases managed with and without CPB.

## 2. Materials and Methods

After institutional review board approval, we retrospectively reviewed the records of 57 patients with RCC and level III or IV caval thrombi who underwent open radical nephrectomy and IVC thrombectomy. The study cohort included patients treated at Duke University Medical Center (Durham, NC, USA) between January 2000 and December 2023. Pathologic staging was determined utilizing the 2009 AJCC TNM staging criteria, and patients treated prior to this were reclassified per criteria. Tumor thrombus level was determined per Mayo classification system [12] and confirmed on pre-operative cross-sectional imaging and/or intraoperative transesophageal echocardiography.

**Figure 1:**
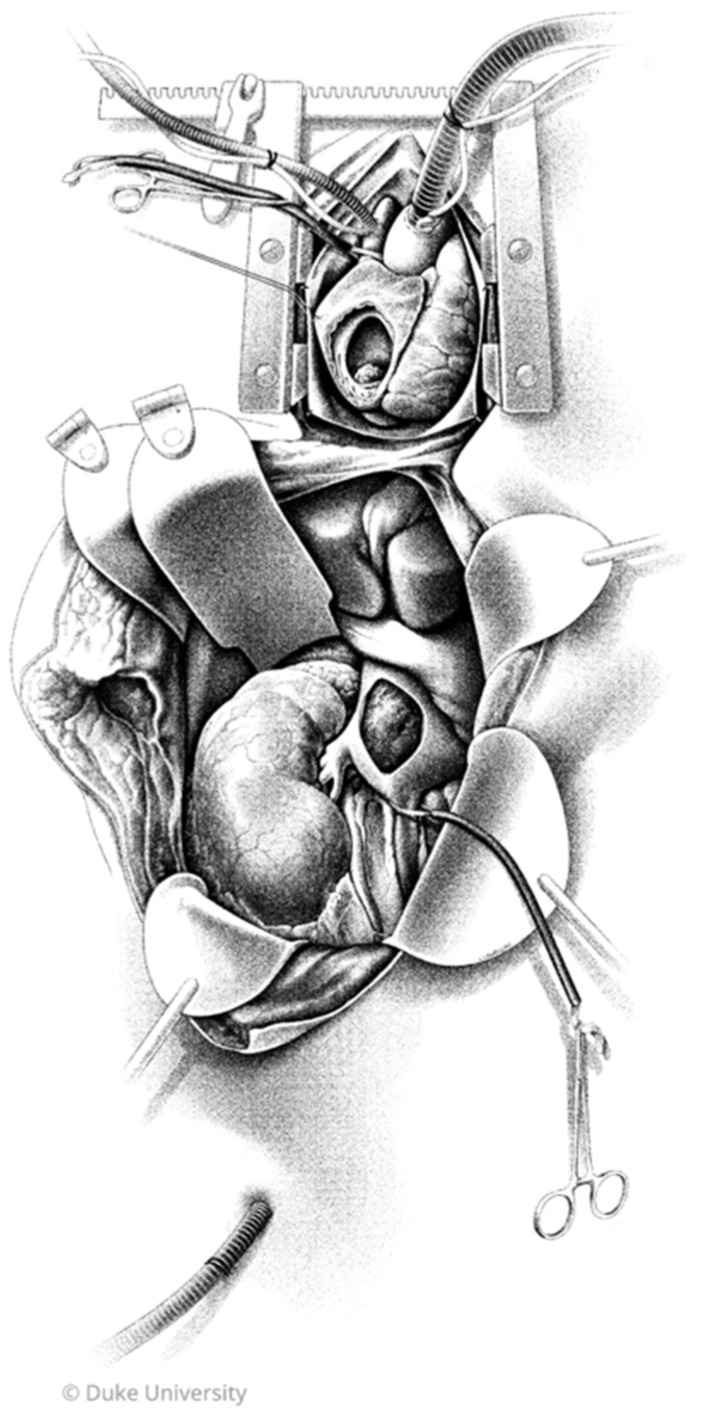
Operative exposure for resection of a right renal cell carcinoma with tumor thrombus (TT) extension to right atrium. A Cattell–Braasch maneuver exposes the right kidney and IVC prior to initiating CPB. Central aortic cannulation and bicaval venous cannulation via peripheral femoral vein and superior vena cava are used for CPB. The SVC and IVC below the TT are snared. Inferior vena cavotomy and right atriotomy are performed to expose and remove the TT en bloc with radical nephrectomy. TT: tumor thrombus; RA: right atrium; IVC: inferior vena cava; CPB: cardiopulmonary bypass; SVC: superior vena cava. Illustrated by Megan Llewellyn, MSMI; copyright Duke University; with permission under a CC BY-ND 4.0 license.

Use of CPB was determined on a case-by-case basis by a multidisciplinary team, and all CPB cases were performed jointly with both cardiothoracic suregeons and urologic oncologists. In total, cases represented patients from eight urologic oncologists and four cardiothoracic surgeons. Technique for CPB was as follows: After exposure of the kidney and intrabdominal IVC, including hepatic mobilization, a median sternotomy was made. Central aortic or axillary arterial cannulation was performed for inflow, and bicaval venous cannulation via peripheral femoral vein and superior vena cava was then performed for outflow. The SVC and IVC below the tumor thrombus are snared, and the patient is then placed on cardiopulmonary bypass. Resection of the tumor thrombus is performed via right atriotomy and vena cavotomy without aortic cross-clamp. A graphic depicting our typical exposure is provided in Figure 1. Deep hypothermic circulatory arrest (DHCA) was not utilized in any of the CPB cases. Operative technique for non-CPB cases was by surgeon preference; no patients were placed on venovenous bypass in our cohort.

Pre-operative angioembolization and regional lymphadenectomy were performed at surgeon discretion. Follow-up intervals were at the discretion of the treatment team and consisted of interval physical exams, laboratory testing, and cross-sectional imaging. Surgical complications were recorded within 90 days and classified with the Clavien–Dindo grading system and further grouped into low-grade (Clavien I–II) and high-grade (Clavien III–IV) complications [13].

Demographic and clinical variables included age, sex, body mass index (BMI), smoking status (current/former), pre-operative hemoglobin, pre-operative serum creatinine, estimated glomerular filtration rate (eGFR), Charlson Comorbidity Index, IVC thrombus level, tumor laterality, pre-operative embolization, metastasis on pre-operative imaging, year of surgery, CPB use, operative time in minutes, estimated blood loss (EBL), intraoperative blood product transfusion, length of hospital stay after index operation (days), tumor size, pathologic staging, 90-day post-operative complication rate, and 90-day mortality. The primary outcome was rates of overall and high-grade ninety-day complications between the CPB and non-CPB groups. The secondary outcome was comparison of overall survival between groups.

Clinical variables and peri-operative outcomes were compared between the CPB and non-CPB groups using the Wilcoxon rank-sum test for continuous variables and the Pearson chi-square test or Fisher’s exact test for categorical variables. Median follow-up was estimated with the reverse Kaplan–Meier method. Overall survival was compared between groups with the Kaplan–Meier (log-rank) method. Cox proportional hazards models with Firth’s penalized likelihood method were used to assess factors associated with overall survival. Firth’s penalized logistic regression was used to assess clinical factors associated with 90-day surgical complications. Candidate variables for multivariable models were selected based on clinical relevance and *p* < 0.10 on univariable analysis. CPB utilization was included in final models regardless of univariate results. All p-values reported were two-sided, and a *p* < 0.05 was considered statistically significant.

Analyses were performed using R version 4.5.1 with RStudio 2025.5.1.513 with the following packages installed: coxphf, ggplot2, ggpubr, ggsurvfit, gtsummary, logistf, lubridate, MASS, tidyverse, tableone, survival, survminer.

## 3. Results

A total of 57 patients were included, with 30 (53%) in the CPB group and 27 in the non-CPB group. Overall, 36 patients had level III and 21 had level IV IVC thrombi. Pre-operative characteristics are summarized in Table 1. All patients in the non-CPB group had a level III thrombus, whereas 9 of 30 (30%) of the CPB cohort had a level III thrombus and 21 of 30 (70%) had a level IV thrombus (*p* < 0.001). The proportion of patients with a Charlson Comorbidity Index score ≥ 1 was higher in the non-CPB cohort (56% vs. 27%; *p* = 0.03). The majority (80%) of CPB patients were treated between 2012 and 2023, compared to 52% of the non-CPB cohort (*p* = 0.049). No other demographic or pre-operative characteristic showed a statistically significant difference between groups.

Intraoperative variables, pathologic outcomes, and complication rates are summarized in Table 2. Median operative time was longer in the CPB group (466 min) compared with the non-CPB group (317 min; *p* < 0.001). Transfusion requirements were similar between groups (median 2200 mL vs. 1400 mL; *p* = 0.2). No intraoperative deaths occurred.

Within 90 days post-surgery, the overall complication and mortality rates were 49% and 10.5%, respectively. Compared with non-CPB patients, those undergoing CPB showed no statistically significant difference in 90-day mortality (10% vs. 11%; *p* = 1.0) or in overall 90-day complications (50% vs. 48%; *p* = 1.0), including low-grade (27% vs. 26%) and high-grade (23% vs. 22%) complications. Counts of complications stratified by afflicted system are available in Table S1.

**Figure 2:**
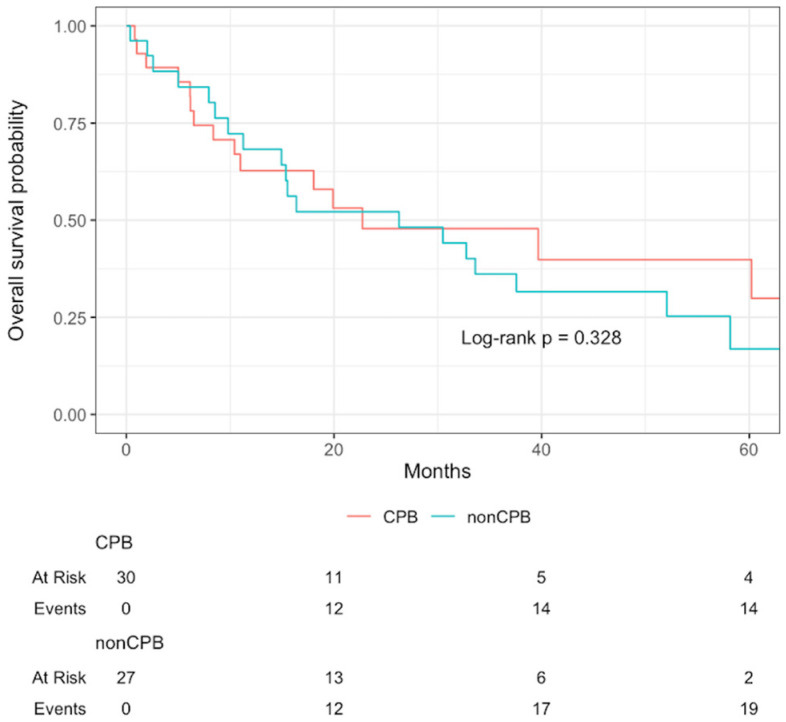
Kaplan–Meier survival curves stratified by CPB use (univariate).

Overall median follow-up, estimated by reverse Kaplan–Meier, was 54.7 months (IQR 33.0–87.7). Three-year overall survival was 47.8% for patients undergoing CPB and 36.1% for those without CPB; the corresponding 5-year survival was 39.8% versus 16.9%, respectively. These differences were not statistically significant (*p* = 0.33), and Kaplan–Meier survival estimates are shown in Figure 2.

On univariate Cox analysis, clinical metastasis, pre-operative hemoglobin, pre-operative creatinine, and pathologic T stage were each significantly associated with increased hazard of mortality and included in the final multivariable model. Only pre-operative metastasis (hazard ratio [HR] 2.31, 95% CI: 1.05–4.93, *p* = 0.039) was found to be an independent predictor for increased hazard of mortality. Use of CPB was not associated with an increased hazard of mortality (HR 1.34, 95% CI 0.65–2.78, *p* = 0.41) (Table 3). This analysis was then repeated to include only patients treated between 2012 aand 2023, and no statistically significant association between OS and CPB use was seen (Table S2).

In analyses of 90-day complications, univariable logistic regression identified higher pre-operative serum creatinine and longer operative time as significant predictors. The final multivariable model included pre-operative serum creatinine, operative time, and CPB use. CPB was not independently associated with increased odds of a 90-day complication (odds ratio [OR] 0.55, 95% CI 0.13–2.12, *p* = 0.4). Longer operative time remained a significant predictor (OR 1.01, 95% CI: 1.0001–1.01, *p* = 0.03) (Table 4). A separate analysis was completed, including only patients treated between 2012 and 2023, and no statistically significant association between CPB use and post-operative complications was found (Table S3).

## 4. Discussion

Managing level III/IV tumor thrombus is one of the greatest challenges in urologic oncology, with continued debate over the risks and benefits of CPB. Concerns over the morbidity and mortality risks of CPB have led to non-CPB management strategies for hemodynamic control in high-level thrombectomy, including venovenous bypass [14,15], liver transplantation techniques, and intrapericardial control [10,11,16]. Though these alternatives to CPB have demonstrated success, it is unclear if CPB is associated with increased risk in RCC with higher-level thrombi. Our findings add to the evidence that CPB is not independently associated with increased complications or mortality in nephrectomy with upper-level thrombectomy.

Previous reports have called into question the risk of CPB’s impact on outcomes. Ngyeun et al. analyzed 362 patients with level III/IV tumor thrombus, comparing outcomes with and without CPB, and found no differences in complication or survival rates based on CPB use, consistent with our results [8]. Our observed high-grade complication rate of 22% aligns with prior reports of 19.5–45% [8,10,17,18,19]. We additionally did not find CPB to be associated with complication rates on multivariable analysis, in line with prior reports [5,8,20]. These findings are contrasted by work from Abel et al., who, in a multicenter study of 162 patients undergoing nephrectomy with upper-level thrombectomy, found CPB to be associated with major complications on univariate analysis (OR 2.16, 95% CI: 1.11–4.22; *p* = 0.02), though they did not include CBP in multivariable analysis [17]. Huang et al. have described a technique for robotic intrapericardial control for level IV tumor thrombi not involving the atria and reported a non-statistically significant lower rate of high-grade complication in their non-CPB group, though in a limited CPB-free sample (*n* = 8) [16]. A recent study by Cai et al. evaluated post-operative neurologic outcomes of patients undergoing nephrectomy with tumor thrombectomy and did not find CPB to be associated with post-operative cognitive impairment [21]. Our findings add to the growing body of evidence that for high-level tumors, CPB is not independently associated with peri-operative complications. These findings persisted when examining patients treated in the contemporary era, providing novel insight given the more historic cohorts reported in prior work.

While higher-level thrombus has been shown to be associated with worse survival outcomes, the contribution of CPB, if any, is undetermined. In multiple previous reports assessing survival outcomes, utilization of CPB was not associated with overall or cancer-specific survival for patients with level III/IV thrombus [5,8,15]. We found similar rates of 90-day mortality among our CPB and non-CBP cohorts (10% vs. 11%) and did not find CPB to be a predictor of mortality on either univariate or multivariate analysis. While not reaching statistical significance, we interestingly did see increased overall survival in our CPB cohort. Larger studies are needed, but this potentially could be related to pre-operative fitness for CPB or intensive intraoperative and post-operative monitoring of patients who went on CPB.

Advancements in CPB, including improvements in neurologic and hemodynamic monitoring, have been well described [22]. Shuch et al. demonstrated that deep hypothermic circulatory arrest (DHCA) was protective against perioperative mortality in patients with level IV thrombus (HR 0.13, 95% CI: 0.036–0.510; *p* = 0.003), though perioperative mortality in their non-DHCA group was 37.5%, notably higher than mortality rates found in our study [6]. These discrepancies may be explained by a more historic cohort, with their study population dating from 1983 to 2007. DHCA was not utilized in our cohort, and further work will be needed to assess its role.

There is no consensus on selecting candidates for CPB or non-CPB approaches for upper-level thrombectomy. Decisions are typically multidisciplinary and institution-dependent. In the case of level III thrombus, some authors advocate only for the use of CPB in patients who do not tolerate IVC clamping and have described non-CPB methods for selected level IV thrombi [23]. Advanced imaging may help elucidate thrombus characteristics pre-operatively and aid in surgical modality selection [24]. Collaboration between the urologic and cardiothoracic surgery teams remains essential. It should be noted that contemporary practice at our center has been to perform cardiac catheterization in all patients potentially undergoing CPB, aiming to optimize surgical candidacy. Given the rarity of these cases, comparative analysis between surgical techniques is limited. Future studies in larger cohorts may be able to directly compare non-CPB modalities both amongst each other and with CPB. Additionally, the role of neoadjuvant systemic immunotherapy with immune checkpoint inhibitors (ICI) prior to nephrectomy and thrombectomy continues to be defined, with a recent series reporting longer operative times but comparable perioperative morbidity among recipients of neoadjuvant ICI compared with a matched cohort of nonrecipients [25]. Further work should clarify the safety of CPB in patients who underwent neoadjuvant immunotherapy, with known increases in systemic inflammation after ICI that potentially could alter surgical outcomes.

Our study is not without limitations, given its nonrandomized, retrospective design and relatively small cohort with limited statistical power. Patients were treated at a single academic tertiary medical center, limiting generalizability, though given the complexity and rarity of these cases requiring multidisciplinary care, outcomes have been shown to be improved at high-volume centers [26]. Despite these limitations, we present contemporary results consistent with prior work, demonstrating that CPB was not independently associated with an increased risk of 90-day perioperative complications, short-term mortality, or overall survival. These findings support individualized decision-making regarding CPB use, informed by perioperative factors and multidisciplinary evaluation.

## 5. Conclusions

Cardiopulmonary bypass was not associated with 90-day postoperative complications or overall survival in our cohort of patients undergoing radical nephrectomy with caval thrombectomy for level III/IV tumor thrombus. Surgical decision-making in this setting is highly complex and multidisciplinary, and future work should focus on identifying optimal candidates for cardiopulmonary bypass.

## Figures and Tables

**Table 1:** Clinical features of patients, stratified by CPB use.

	CPB (*n* = 30)	Non-CPB(*n* = 27)	*p*
Age (median [IQR])	64 [59, 67]	66 [61, 71]	0.1
Sex = Male, *n* (%)	18 (60.0)	20 (74.1)	0.399
BMI (kg/m^2^) (median [IQR])	29.3 [25.5, 32.2]	27.9 [25.5, 30.9]	0.532
Preop Hemoglobin (g/dL), (median [IQR])	9.9 [8.8, 12.4]	11.6 [9.8, 12.7]	0.127
Preop Creatinine (mg/dL), (median [IQR])	1.20 [1.02, 1.30]	1.30 [1.10, 1.90]	0.339
Smoking (current/former), *n* (%)	18 (60)	18 (67)	0.806
CCI, *n* (%)			
0	22 (73)	12 (44)	0.033
≥1	8 (27)	15 (56)	
Laterality, *n* (%)			
Left	12 (40)	9 (33)	0.806
Right	18 (60)	18 (67)	
Thrombus Level, *n* (%)			
3	9 (30)	27 (100)	<0.001
4	21 (70)	0 (0)	
Metastasis, *n* (%)	10 (33)	7 (26)	0.749
Preop Angioembolization, *n* (%)	8 (27)	6 (22)	0.935
Year of surgery, *n* (%)			
2000–2011	6 (20)	13 (48)	0.049
2012–2023	24 (80)	14 (52)	

BMI: body mass index; CBP: cardiopulmonary bypass; CCI: Charlson comorbidity index; IQR: interquartile range.

**Table 2:** Intra-operative variables and peri-operative outcomes stratified by CPB use.

	CPB (*n* = 30)	Non-CPB (*n* = 27)	*p*
Operative time (min), (median [IQR])	466 [386, 512]	317 [271, 405]	<0.001
Intraop Transfusion (mL), (median [IQR]) *	2300 [1300, 3400]	1400 [0, 2863]	0.193
Pathologic size (cm), (median [IQR])	12.0 [10.0, 13.8]	10.6 [7.6, 15.0]	0.397
Pathologic T stage, *n* (%)			
T3	28 (93)	24 (89)	0.902
T4	2 (7)	3 (11)	
Pathologic N stage, *n* (%)			
N0/Nx	23 (77)	24 (89)	0.388
N1	7 (23)	3 (11)	
90-day post-op complications	15 (50)	13 (48)	1
Low (Clavien 1–2)	8 (53)	7 (54)	
High (Clavien 3–4)	7 (47)	6 (46)	
LOS (days), (median [IQR])	8.5 [7.0, 13.0]	8.0 [6.0, 10.5]	0.193
90-day mortality, *n* (%)	3 (10)	3 (11)	1

CPB: Cardiopulmonary bypass; EBL: estimated blood loss; IQR: interquartile range; LOS: length of stay. * Cell saver was used in some patients. Transfusion was calculated from blood/blood products.

**Table 3:** Multivariable Cox proportional hazards model with Firth’s penalized likelihood for predictors of mortality.

	HR	95% CI	*p*
CPB			
No	-	-	
Yes	1.34	0.65, 2.78	0.41
Pre-op Hgb (g/dL)	0.88	0.75, 1.05	0.17
Metastasis	2.31	1.05, 4.93	0.038
pTstage			
T3	-	-	
T4	2.5	0.83, 7.54	0.1
Pre-op sCr (mg/dL)	1.38	0.99, 1.83	0.54

CI: confidence interval; CPB: cardiopulmonary bypass; HR: hazard ratio; Hgb: hemoglobin (g/dL); sCr: serum creatinine.

**Table 4:** Multivariable Firth’s penalized logistic regression for variables associated with 90-day complication.

	OR	95% CI	*p*
CPB			
No	-	-	
Yes	0.55	0.13, 2.12	0.4
Operative Time	1.01	1.0001, 1.01	0.030
Pre-operative Creatinine	1.59	0.86, 4.77	0.14

CI: confidence interval; CPB: cardiopulmonary bypass; OR: odds ratio.

## Data Availability

The datasets presented in this article are not readily available because of the protection of personal health information, with the dataset stored behind an institutional firewall. Requests to access the datasets should be directed to the corresponding author.

## Supplementary Materials

The following supporting information can be downloaded at https://www.mdpi.com/article/10.3390/curroncol32120671/s1, Table S1: Post-operative complications stratified by CPB use. Table S2. Multivariable Cox proportional hazards model with Firth’s penalized likelihood for predictors of mortality for patients treated from 2012 to 2023. Table S3: Multivariable Firth’s penalized logistic regression for variables associated with 90-day complication for patients treated from 2012 to 2023.

## Abbreviations

The following abbreviations are used in this manuscript:

BMI	Body Mass Index
CCI	Charlson Comorbidity Index
CPB	Cardiopulmonary Bypass
IVC	Inferior Vena Cava
HR	Hazard Ratio, OR: Odds Ratio
RCC	Renal Cell Carcinoma
SVC	Superior Vena Cava
