# Supplementary material for: Does Cardiopulmonary Bypass Affect Outcomes in Nephrectomy with Level III/IV Caval Thrombectomy for Renal Cell Carcinoma?
Source: Curr Oncol. 2025 Nov 29;32(12):671. doi: 10.3390/curroncol32120671 (PMC12731594; doi:10.3390/curroncol32120671)
Supplement: Supplementary file 1 [file curroncol-32-00671-s001.zip › Table_S1.pdf]

Table S1: Post-operative complications stratified by CPB use.

	nonCPB	CPB	p
	32	37	
Arrythmia	1 (6)	3 (14)	0.679
Cardiothoracic	2 (11)	2 (9)	
Gastrointestinal	3 (17)	3 (14)	
Hematologic	3 (17)	3 (14)	
Infectious	2 (11)	3 (14)	
Neurologic	1 (6)	2 (9)	
Pulmonary	3 (17)	2 (9)	
Renal	3 (17)	2 (9)	
Thromboembolic	0 (0.0)	2 (9)	
