# Supplementary material for: Does Cardiopulmonary Bypass Affect Outcomes in Nephrectomy with Level III/IV Caval Thrombectomy for Renal Cell Carcinoma?
Source: Curr Oncol. 2025 Nov 29;32(12):671. doi: 10.3390/curroncol32120671 (PMC12731594; doi:10.3390/curroncol32120671)
Supplement: Supplementary file 1 [file curroncol-32-00671-s001.zip › Table_S2.pdf]

Table S2. Multivariable Cox proportional hazards model with Firth's penalized likelihood for predictors of mortality for patients treated from 2012-2023.

	HR	95% CI	p
CPB			
No	-	-	
Yes	1.85	0.79,4.35	0.15
Pre-op Hgb (g/dL)	0.88	0.75,1.05	0.047
Metastasis	7.71	2.70,22.82	>0.001
pTstage			
T3	-	-	
T4	2.5	0.83,7.54	0.1
Pre-op sCr (mg/dL)	1.11	0.76,1.50	0.56

CI: confidence interval, CPB: cardiopulmonary bypass; HR: Hazard ratio; Hgb: Hemoglobin (g/dL); sCr: serum Creatinine
