# Supplementary material for: Does Cardiopulmonary Bypass Affect Outcomes in Nephrectomy with Level III/IV Caval Thrombectomy for Renal Cell Carcinoma?
Source: Curr Oncol. 2025 Nov 29;32(12):671. doi: 10.3390/curroncol32120671 (PMC12731594; doi:10.3390/curroncol32120671)
Supplement: Supplementary file 1 [file curroncol-32-00671-s001.zip › Table_S3.pdf]

Table S3. Multivariable Firth's penalized logistic regression for variables associated with 90-day complication for patients treated from 2012-2023.

	OR	95% CI	p
CPB			
No	-	-	
Yes	0.35	0.04,2.04	0.3
Operative Time	1.01	1.00,1.02	0.14
Pre-operative Creatinine	1.31	0.70,3.49	0.40

CI: confidence interval, CPB: cardiopulmonary bypass; OR: Odds ratio.
